# Baicalein inhibits cell growth and increases cisplatin sensitivity of A549 and H460 cells via miR‐424‐3p and targeting PTEN/PI3K/Akt pathway

**DOI:** 10.1111/jcmm.13556

**Published:** 2018-02-01

**Authors:** Chunya Lu, Huaqi Wang, Shanshan Chen, Rui Yang, Hui Li, Guojun Zhang

**Affiliations:** ^1^ Department of Respiratory Medicine The First Affiliated Hospital of Zhengzhou University Zhengzhou Henan China

**Keywords:** baicalein, miR‐424‐3p, non‐small‐cell lung cancer, PTEN

## Abstract

Lung cancer is the leading cause of death in individuals with malignant disease. Non‐small‐cell lung cancer (NSCLC) is the most common type of lung cancer, and chemotherapy drugs such as cisplatin are the most widely used treatment for this disease. Baicalein is a purified flavonoid compound that has been reported to inhibit cancer cell growth and metastasis and increase sensitization to chemotherapeutic drugs via different pathways. Therefore, we assessed the effects of baicalein on the proliferation, apoptosis and cisplatin sensitivity in the NSCLC A549 and H460 cell lines and determined the pathways through which baicalein exerts its effects. Baicalein was slightly toxic to normal human bronchial NHBE cells but inhibited growth, induced apoptosis and increased cisplatin sensitivity in A549 and H460 cells. Baicalein down‐regulated miR‐424‐3p, up‐regulated PTEN expression and down‐regulated expression of PI3K and p‐Akt in A549 and H460 cells. Dual‐luciferase reporter assay demonstrated that PTEN is a target gene of miR‐424‐3p, and overexpression of miR‐424‐3p or silencing of PTEN partially attenuated the effects of baicalein on A549 and H460 cells. Taken together, we concluded that baicalein inhibits cell growth and increases cisplatin sensitivity to A549 and H460 cells via down‐regulation of miR‐424‐3p and targeting the PTEN/PI3K/Akt pathway.

## 1 INTRODUCTION

Lung cancer is currently the leading cause of death in individuals with malignant disease worldwide,^1^, ^2^ with a 5‐year survival rate of less than 20%.^3^ Non‐small‐cell lung cancer (NSCLC) is the most common type of lung cancer, accounting for approximately 85% of all lung cancers. Lung squamous cell carcinoma and lung adenocarcinoma are the most common types of NSCLC.^4^ The proportion of patients that undergo surgical treatment for NSCLC is quite small, as most cases are often diagnosed at an advanced stage. In addition, although molecular‐targeted therapy plays an important role in NSCLC therapy, chemotherapy drugs such as cisplatin are still the classic and most common treatment.^5^ However, poor responses and individual differences occur during cisplatin treatment, and the incidence of intrinsic or acquired resistance is high.^6^, ^7^ Therefore, it is particularly important for researchers to discover novel anticancer or chemotherapy‐sensitizing agents.

**Figure 1:**
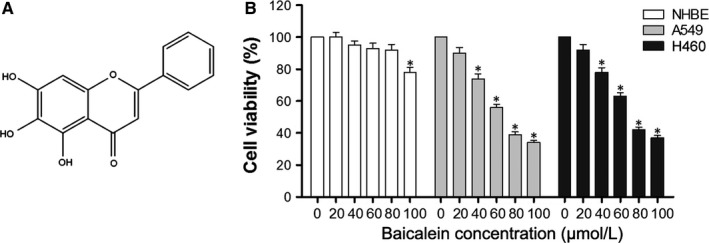
Cytotoxic effects of baicalein in A549, H460 cells and NHBE cells. (A) Chemical structure of baicalein. (B) NHBE, A549 and H460 cells were treated with different concentrations of baicalein for 24 h, and CCK‐8 was used to detect cell viability of three cell lines. *P < .05

Baicalein, a purified flavonoid compound also known as 5,6,7‐trihydroxyflavone (Figure 1A), is a Chinese herbal medicine extracted from the dry roots of the *Scutellaria baicalensis* plant. Baicalein has been reported to exhibit potential anticancer effects in many studies.^8^, ^9^ In addition to lung cancer, baicalein also inhibits the growth and metastasis of prostate cancer cells,^10^ the invasion of gastric cancer cells,^11^ the migration, adhesion and invasion of breast cancer cells,^12^ and induces apoptosis and autophagy in hepatocellular carcinoma cells.^13^, ^14^ In addition, some studies have demonstrated the effects of baicalein on cisplatin sensitivity via different pathways in various cancer cells.^15^, ^16^, ^17^ Baicalein has also exhibited a wide range of anti‐inflammatory effects associated with airway injury, liver injury and rheumatoid arthritis.^18^, ^19^, ^20^ In summary, baicalein has the potential to become an ideal adjuvant therapy in the treatment of cancer.

Previous studies of baicalein have identified several pathways, such as the ROS/AMPK pathway in lung cancer,^21^ the caveolin‐1/Akt/mTOR pathway in prostate cancer,^10^ the p38 signalling pathway,^11^ the PTEN/Akt/HIF‐1α signalling pathway^22^ in gastric cancer, the NF‐κB signalling pathway in ovarian cancer,^23^ the Wnt/β‐catenin pathway in breast cancer,^24^ the PI3K/Akt pathway in oesophageal squamous cell carcinoma^25^ and the ERK pathway in hepatocellular carcinoma.^26^ Phosphatase and tensin homolog (PTEN) is known as a tumour suppressor gene that has been reported to affect cancer cell behaviour in various cancers.^27^, ^28^, ^29^, ^30^ PTEN may inhibit cellular proliferation, growth and survival through the PI3K/Akt/mTOR pathway.^31^, ^32^ There is also evidence that baicalein may exert its effects via the PTEN/Akt pathway.^22^, ^33^

MicroRNAs (miRNAs) are small non‐coding RNAs with lengths of 19‐25 nucleotides that regulate the translation or degradation of target mRNA in the human body.^34^ MiRNAs play an important role in the proliferation, invasion and apoptosis of malignant tumour cells and may affect resistance to cisplatin.^35^, ^36^, ^37^ In addition, pathways that link PTEN and miRNAs exist in cancers.^38^, ^39^, ^40^, ^41^

In this study, we assessed the effects of baicalein on proliferation, apoptosis and cisplatin sensitivity in NSCLC A549 and H460 cell lines. We also determined changes in miRNA expression caused by baicalein treatment using the miRNA microarray and validated the hypothesis that baicalein affects the miRNA‐PTEN/PI3K/Akt pathway. Our study indicated that baicalein may inhibit cell growth and increase cisplatin sensitivity to A549 and H460 cells by down‐regulating miR‐424‐3p and targeting the PTEN/PI3K/Akt pathway.

## 2 MATERIALS AND METHODS

### 2.1 Cells and reagents

Non‐small‐cell lung cancer A549 and H460 cell lines and the normal human bronchial epithelial (NHBE) cell line were purchased from the Cell Bank of Shanghai Institutes for Biological Sciences of the Chinese Academy of Sciences (Shanghai, China); baicalein (465119) and dimethyl sulphoxide (DMSO, D2650) were purchased from Sigma‐Aldrich (St. Louis, MO, USA). Cisplatin was purchased from the Qilu Pharmaceutical Company (Jinan, Shandong, China). Primary antibodies for Western blotting against to PTEN (ab32199), survivin (ab469), Bcl‐xL (ab32370) and β‐actin (ab8227) was purchased from Abcam (Cambridge, UK); antibodies for PI3K (SAB5500162), total Akt (t‐Akt, SAB4500797) and phosphor‐forms (p‐Akt, SAB4301414) were purchased from Sigma‐Aldrich; the secondary antibody (ab205718) for Western blotting was purchased from Abcam (Cambridge, UK).

### 2.2 Cell culture

Cells were cultured in an incubator (Thermo Fisher Scientific, Waltham, MA, USA) in a humidified atmosphere with 5% CO_2_ at 37°C. All cells were cultured in high‐glucose Dulbecco's modified Eagle's medium (DMEM; Gibco, Waltham, MA, USA) with 10% foetal bovine serum (FBS; Gibco) and 1% penicillin‐streptomycin (Gibco). Trypsin (0.25%) (Gibco) was used to dissociate cells. Different concentrations of baicalein or DMSO (vehicle control) were added to the cells during experiments.

### 2.3 Cell transfection

The miR‐424‐3p mimics, miR‐424‐3p inhibitor, miR‐scramble or si‐PTEN (Shanghai GenePharma Co., Ltd., Shanghai, China) were transfected, or cotransfected, into cells according to the experimental design. When cell confluence was approximately 50%‐70%, the medium was replaced with serum‐free medium, and transfection was performed in 6‐well plates (Corning, Corning, NY, USA) using Lipofectamine™ 2000 (Invitrogen, Carlsbad, CA, USA) per manufacturer's recommendations. Six hours after transfection, medium was changed to medium plus FBS; cells were harvested for assays 24‐72 hours after transfection.

### 2.4 RNA extraction and quantitative RT‐PCR

Total RNA in cells was extracted using TRIzol (Invitrogen, Waltham, CA, USA), and assessment of RNA concentration and quality was performed by Nanodrop 2000 spectrophotometry (Thermo Fisher Scientific). Quantitative real‐time reverse transcription‐polymerase chain reaction (qRT‐PCR) was performed to determine the relative expression levels of miR‐424‐3p, miR‐377‐3p and miR‐1224‐5p using the 7500 Fast PCR instrument (Applied Biosystems, Waltham, MA, USA). GAPDH (Invitrogen, Waltham, MA, USA) was used as an internal control for miRNAs. After total RNA extraction, a First Strand cDNA Synthesis Kit (Thermo Scientific, Waltham, MA, USA) was used to generate cDNA; qRT‐PCR was then performed with SYBR Premix Ex TaqII (TaKaRa, Japan). Relative miRNA expression levels were calculated by the 2^−ΔΔCt^ method. Primer sequences were synthesized by Shanghai Biological Technology Company (Shanghai, China).

### 2.5 Protein extraction and Western blotting

Cells were lysed using RIPA lysis buffer (Solarbio, Beijing, China) according to manufacturer's instructions. The BCA protein assay kit (Beyotime, Shanghai, China) was used to determine protein concentration of the samples. Samples were then subjected to sodium dodecyl sulphate‐polyacrylamide gel electrophoresis (SDS‐PAGE) and transferred onto a polyvinylidene difluoride filter (PVDF) membrane (Thermo Scientific). The membrane was blocked with 5% skim milk powder for 1 hour and then washed four times with Tris‐buffered saline containing Tween 20 (TBST; Solarbio, Beijing, China). The PVDF membrane was then incubated overnight at 4°C with primary antibodies for PTEN, survivin, Bcl‐xL, t‐Akt, p‐Akt, PI3K and β‐actin (endogenous control), at a concentration of 1:1000. Following incubation, membranes were washed with TBST and incubated with secondary goat anti‐rabbit antibody for 1 hour at room temperature, at a concentration of 1:10 000. Membranes were again washed with TBST, and protein immunoreactivity was determined by FluorChem E (ProteinSimple, San Jose, CA, USA) using the ECL Western Blotting Substrate (Thermo Scientific). Quantified data were analysed using IPP image analysis software.

### 2.6 Cell counting kit‐8 assay

The cell counting kit‐8 (CCK‐8; Dojindo, Kumamoto, Japan) assay was used to examine the viability of cells following different treatments. Cells were seeded in a 96‐well plate (100 μL) at a density of 4 × 10^5^/mL and treated with different concentrations of baicalein and cisplatin, or transfected with miRNA mimics/scramble or siRNA when the cell confluence was approximately 50%‐70%. After 24‐72 hours, the medium was replaced by CCK‐8 solution (10 μL) and DMEM with FBS (90 μL) for 1 hour. The 96‐well plates were then put into a microplate reader (Bio‐Rad, Hercules, CA, USA), and the optical density (OD) of each well at 450 nm was measured. Cell viability was calculated using OD levels (cell viability (%) = OD_(treatment group)_/OD_(control group)_ × 100%). Calculation of the half inhibitory concentration (IC50) was calculated using the OD value at 24 hours, in cells cotreated with different concentrations of cisplatin (0, 2, 4, 8, 16, 32 μmol/L). The IC50 represents the cisplatin concentration when cell viability was inhibited 50%.

### 2.7 Clone formation assay

Soft agar clone formation assay was used to measure the colony‐forming ability of cells. Following high‐pressure steam sterilization, 1.2% and 0.7% agarose gels (Low melting gel; Solarbio, Beijing, China) were put into a water bath (55°C) for preparation. Agarose (1.2%) plus an equal volume of 2× DMEM (20% FBS + 2% penicillin‐streptomycin) was added to the lower layer of a 6‐cm dish and allowed to solidify at room temperature. Following transfection, cells were dissociated and suspended in 37°C DMEM plus 20% FBS at a density of 5 × 10^4^/mL. Next, 100 μL of cell suspension (5000 cells) was suspended into a 1:1 solution of 0.7% agarose and 2 ×  DMEM (3 mL total) in the upper layer of growth agar. Different concentrations of baicalein (0, 40 μmol/L) were added into the upper layer of growth agar, and cells were incubated for 14 days. After incubation, cells were fixed with a combination of 10% methanol and 10% acetic acid, and then stained with 1% crystal violet (Solarbio, Beijing, China). The number of colonies containing more than 50 cells was determined using an optical microscope (Olympus, Tokyo, Japan).

### 2.8 Cell apoptosis assays

The Annexin V‐FITC/PI Apoptosis Detection Kit (Solarbio, Beijing, China) was used to detect apoptotic cells. Cells were collected 24 hours after treatment, washed with PBS (Solarbio, Beijing, China) and resuspended in binding buffer (500 μL) in an Eppendorf (EP) tube. Annexin V‐FITC and PI (5 μL) were added into binding buffer. After mixing, the EP tube was kept away from light for 5‐15 minutes at room temperature. Flow cytometry (BD, San Diego, CA, USA) was used to identify cells of normal status, early apoptosis, late apoptosis and death; FITC was detected using channel FL1, and PI was detected using channel FL3.

Caspase‐3/7 activity was measured using the Apo‐ONE^®^ Homogeneous Caspase‐3/7 Assay kit (Promega, Madison, WI, USA). Cells were seeded in 96‐well plates and cultured with baicalein (0, 40, 80 μmol/L) for 24 hours. Apo‐ONE^®^ Caspase‐3/7 reagent was added (100 μL) and mixed with medium, and the plate covered with a plate sealer and incubated for 3 hours. The fluorescence (RFU) of each well was measured using a spectrofluorometer (Thermo Scientific) at an excitation wavelength of 499 nm and an emission wavelength of 521 nm. Caspase‐3/7 activity = RFU_(treatment group)_/RFU_(control group)_ × 100%.

### 2.9 miRNA microarray assay

Cells were treated with either DMSO (vehicle control) or 40 μmol/L baicalein (three duplicates per group) for 24 hours, total RNA was extracted, and the miRNA microarray assay conducted. RNA integrity was assessed using the Agilent Bioanalyzer 2100 (Agilent Technologies, Santa Clara, CA, USA). The SurePrint Human microRNA microarray (Shanghai Bohao Biotechnology Co., Ltd, Shanghai, China) was used for detection and data analysis. The labelling, microarray hybridization and washing of samples were performed per manufacturer's protocols. After washing, arrays were scanned using the Agilent Scanner G2505C (Agilent Technologies). Feature Extraction software (version 10.7.1.1, Agilent Technologies) was used to analyse array images and obtain raw data. GeneSpring (version 13.1, Agilent Technologies) was used to analyse the basic raw data. Differentially expressed miRNAs were identified through fold change, and *P*‐values were calculated using Student's *t* test. The threshold set for differential expression was a fold change of ≥2.0 and a *P*‐value of ≤.05. Hierarchical clustering was performed to display distinguishable miRNA expression patterns among samples. The differentially expressed miRNAs in NSCLC A549 and H460 cells obtained from the miRNA microarray were validated using qRT‐PCR.

### 2.10 Dual‐luciferase reporter assay

Dual‐luciferase reporter assay was used to determine whether PTEN is a target gene of miR‐424‐3p. The pmirGLO vector was purchased from Promega (Madison, WI, USA). Recombinant vectors containing wild‐type (pmirGLO‐Wt‐PTEN 3′UTR) and mutant‐type (pmirGLO‐Mut‐PTEN 3′UTR) PTEN mRNA 3′UTR were constructed by Shanghai Biological Technology Company (Shanghai, China). miR‐424‐3p mimics or miR‐scramble were cotransfected with pmirGLO‐Wt‐PTEN 3′UTR or pmirGLO‐Mut‐PTEN 3′UTR into A549 and H460 cells, creating four test groups. Luciferase activity was determined 24 hours after cotransfection, using a luciferase assay kit (Promega, Madison, WI, USA) on a luminescence microplate reader (Berthold, Bad Wildbad, Germany).

### 2.11 Animal experiment

This study was approved by the Medical Research Ethics Committee of The First Affiliated Hospital of Zhengzhou University. A xenograft mouse model was used to assess the effects of baicalein on NSCLC cells in vivo. Female nude mice (n = 20, BALB/c, 4‐6 weeks old, 16‐18 g) were purchased from the Beijing Vital River Laboratory Animal Technology Center (Beijing, China). Mice were housed in a facility at a constant temperature and supplied with laboratory chow and water. Bedding was changed once a week, and the mice were kept on a 12‐hour light/dark cycle. Treatment of the mice was carried out under sterile conditions in a microbiological safety cabinet. The A549 cell line with firefly luciferase stably expressed (A549‐luc) was obtained from PerkinElmer (Waltham, MA, USA). Mice were implanted with 5 × 10^6^ A549‐luc cells in 0.2 mL into the left armpit. One week after implantation, the tumour nodules were visible, and mice were randomly divided into 4 groups: vehicle control (20% DMSO in PBS), baicalein, cisplatin and baicalein combined with cisplatin. Baicalein or vehicle control was intraperitoneally injected (3 mg/kg) daily, and cisplatin was injected (3 mg/kg) twice weekly. To detect tumour growth via luciferase signal, mice were observed weekly in an in vivo small animal imaging instrument (PerkinElmer, Waltham, MA, USA). D‐luciferin sodium salt (Sigma‐Aldrich) (10 μL/g) was injected intraperitoneally into the mice 10 to 15 minutes before detection. Mice were killed 4 weeks after implantation, and tumours were cut out and weighed.

### 2.12 Statistical analysis

Each experiment was repeated three times. Statistical analysis was performed with SPSS 21 software, and data were expressed as mean ± standard deviation (S.D.). One‐way ANOVA was carried out to compare three or more groups; Student's *t* test was used to compare two independent groups. The IC50 of cisplatin was calculated using the normal probability conversion method and probit regression analysis. A *P*‐value of <.05 was considered statistically significant.

## 3 RESULTS

### 3.1 Baicalein exerts different cytotoxic effects in NHBE cells and NSCLC A549 and H460 cells

We used the CCK‐8 assay to determine the cytotoxic effects of baicalein at different concentrations (0, 20, 40, 60, 80, 100 μmol/L) in NHBE cells and NSCLC A549 and H460 cells. As shown in Figure 1B, a dose‐dependent cytotoxic effect of baicalein was clearly shown in A549 and H460 cells, whereas the NHBE cells were largely unaffected. This demonstrates that NSCLC and NHBE cells had differing responses to baicalein treatment. The viability of A549 and H460 cells was significantly inhibited by baicalein, whereas in NHBE cells, there was no significant cytotoxic effect.

### 3.2 Baicalein inhibits cell proliferation, promotes apoptosis and increases cisplatin sensitivity in A549 and H460 cells via up‐regulation of PTEN and suppression of the PI3K/Akt pathway

**Figure 2:**
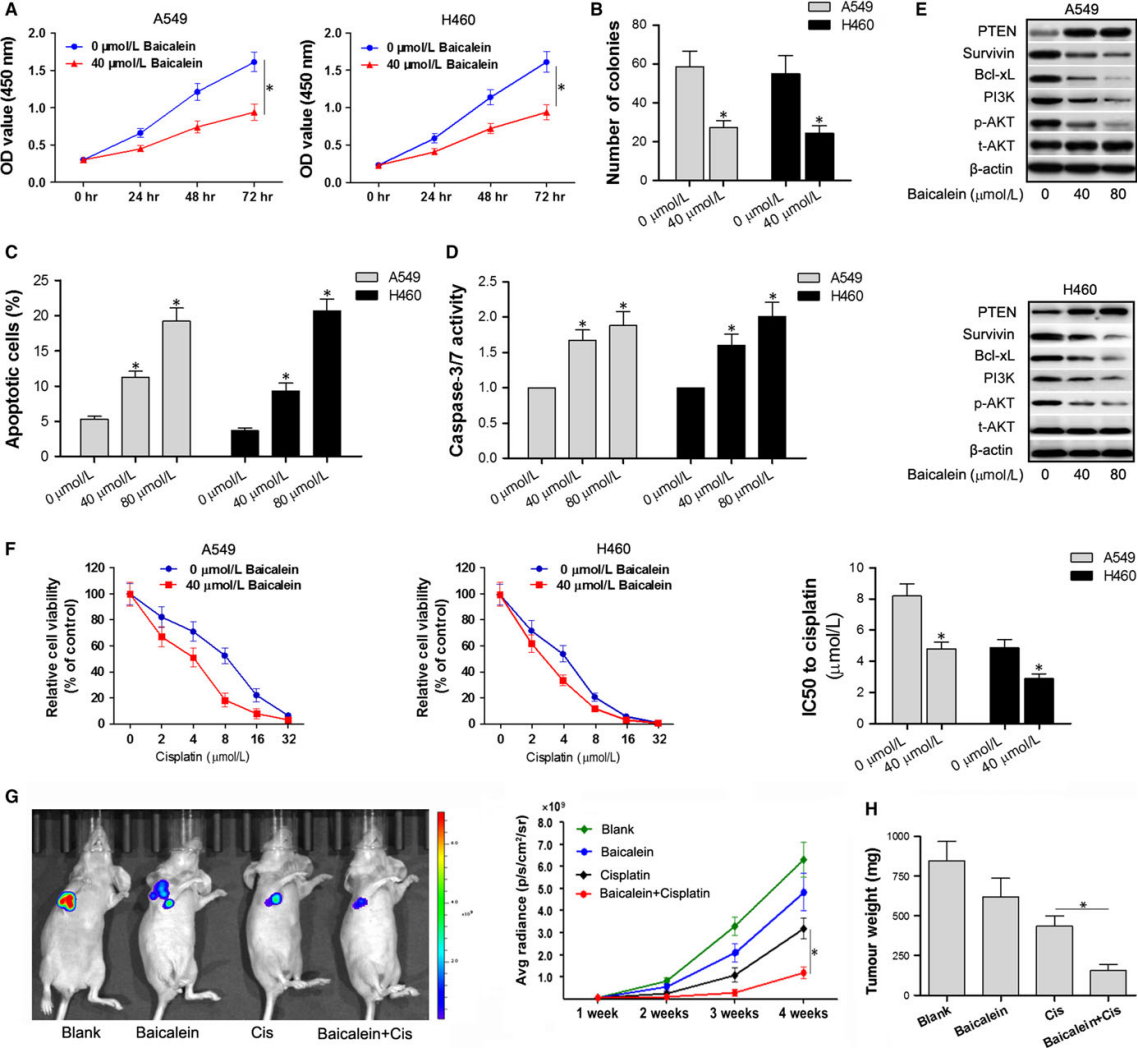
Baicalein inhibits cell proliferation, promotes apoptosis and increases cisplatin sensitivity in A549 and H460 cells via up‐regulation of PTEN and suppression of the PI3K/Akt pathway. (A) A549 and H460 cells were treated with 0 or 40 μmol/L baicalein for 0‐72 h, and CCK‐8 was performed to measure cell proliferation. (B) Clone formation assay was used to detect number of colonies 24 h after baicalein treatment. (C) Annexin V‐FITC/PI double staining and flow cytometry were used to detect apoptosis in A549 and H460 cells treated with 0, 40, 80 μmol/L baicalein for 24 h. (D) Caspase‐3/7 activity assay kit was used to detect caspase‐3/7 activity in A549 and H460 cells. (E) Western blotting was performed to detect expression levels of survivin, Bcl‐xL and proteins involved in the PTEN/PI3K/Akt pathway 48 h after baicalein treatment. (F) Cells treated with 0 or 40 μmol/L baicalein were cotreated with different concentrations of cisplatin for 24 h, CCK‐8 was used to detect cell viability, and the IC50 was calculated. IC50 indicates the concentration of cisplatin at which cell viability is inhibited by 50%. (G,H) Xenograft mice were divided into four groups: vehicle control, baicalein, cisplatin and baicalein combined with cisplatin. Average radiance of each mouse was observed weekly, and tumour weights were recorded at week 4. *P < .05

To evaluate the antiproliferative effects of baicalein, A549 and H460 cells were treated with 0 or 40 μmol/L baicalein for up to 72 hours. The proliferation of A549 and H460 cells was significantly inhibited by baicalein after 24, 48 and 72 hours (*P* < .05) (Figure 2A,B). Moreover, baicalein induced apoptosis and increased caspase‐3/7 activity in A549 and H460 cells, in a dose‐dependent manner (Figure 2C,D) (*P* < .05). As shown in Figure 2F, the combination of baicalein and different concentrations of cisplatin (0, 2, 4, 8, 16, 32 μmol/L) resulted in greater inhibition of cell viability in A549 and H460 cells than cisplatin alone (*P* < .05). In addition, baicalein treatment increased cisplatin sensitivity, as is shown by the lower IC50 (*P* < .05). To further confirm the effect of baicalein on cisplatin sensitization in vivo, the A549 xenograft model was used (Figure 2G). Results showed that the average radiance in xenograft mice treated with cisplatin plus baicalein was significantly lower than that of mice treated with cisplatin alone (*P* < .05). Similar results were observed with tumour weights (Figure 2H). Overall, baicalein inhibited proliferation, promoted apoptosis and increased cisplatin sensitization in A549 and H460 cells.

To investigate the potential pathways by which baicalein may induce its effects, we performed Western blotting in A549 and H460 cells treated with 0, 40 and 80 μmol/L baicalein, and determined the expression levels of proteins involved in proliferation and apoptosis. As shown in Figure 2E, baicalein up‐regulated the expression of PTEN and down‐regulated the expression of survivin and Bcl‐xL. In addition, the expression of PI3K and phosphorylation of Akt were both decreased by baicalein treatment. The above results indicate that baicalein may suppress the PI3K/Akt pathway by up‐regulating PTEN.

### 3.3 Baicalein down‐regulates miR‐424‐3p in A549 and H460 cells

**Figure 3:**
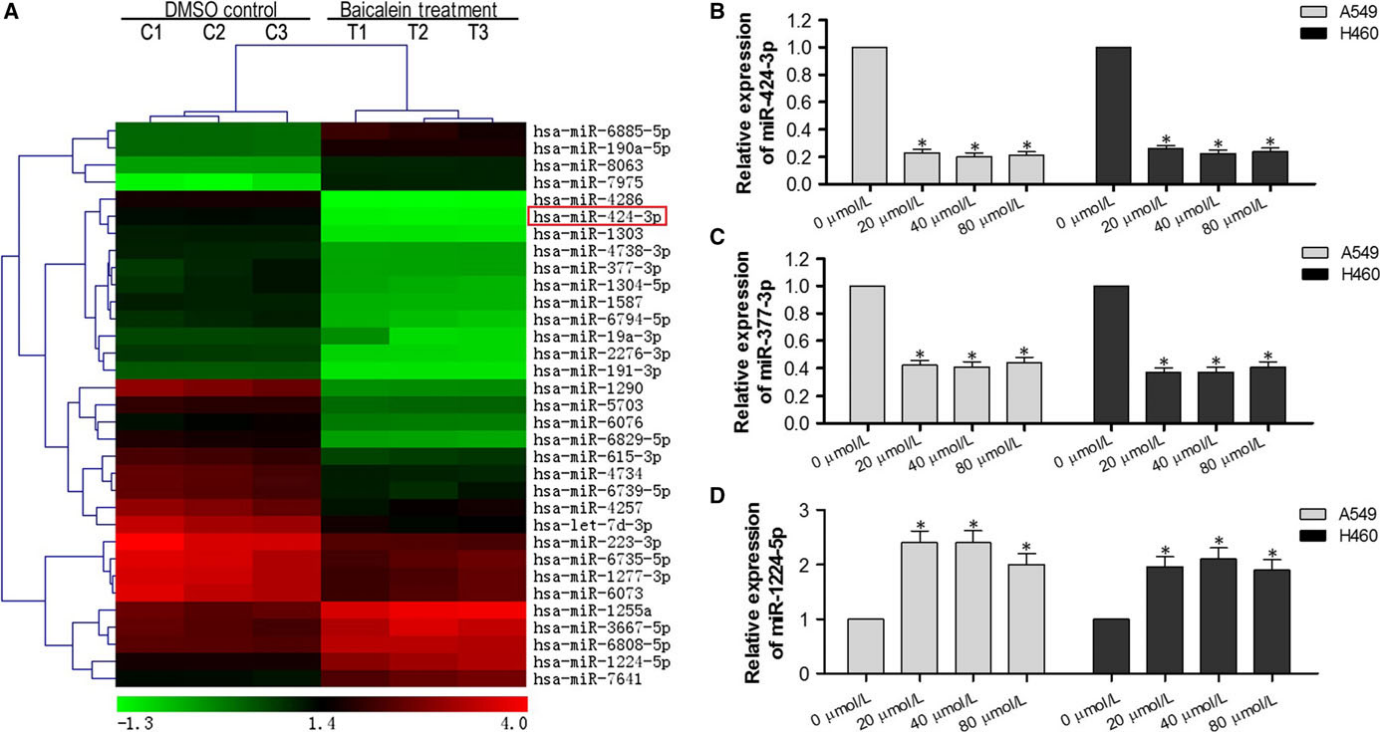
Baicalein down‐regulates miR‐424‐3p expression in A549 and H460 cells. (A) A549 cells treated with DMSO or 40 μmol/L baicalein for 24 h were collected for RNA extraction and miRNAs microarray analysis. Hierarchical clustering was performed to display the differentially expressed miRNAs with a fold change of ≥2.0 and a *P*‐value of ≤.05. (B) qRT‐PCR was performed to validate the differential expression of miR‐424‐3p in NSCLC A549 and H460 cells. (C) qRT‐PCR was performed to validate the differential expression of miR‐377‐3p in NSCLC A549 and H460 cells. (D) qRT‐PCR was performed to validate the differential expression of miR‐1224‐5p in NSCLC A549 and H460 cells. *P < .05

To determine the molecular mechanisms behind the effects of baicalein in NSCLC cells, we performed miRNA microarray analysis in A549 cells treated with different concentrations of baicalein (0, and 40 μmol/L) for 24 hours. As shown in Figure 3A and Table 1 miRNAs were differentially expressed, with a greater than twofold change in cells treated with 40 μmol/L baicalein as compared to DMSO controls (*P* < .05). Of the 33 differentially expressed miRNAs, 24 were down‐regulated and 9 were up‐regulated. Interestingly, the microarray results indicated that miR‐424‐3p was significantly down‐regulated, with a greater than fivefold change.

**Table 1:** Summary of differentially expressed miRNAs by baicalein treatment using microarray

Name	miRNA location	Fold change	Regulation
hsa‐miR‐424‐3p	chrX	5.517	Down
hsa‐miR‐19a‐3p	chr13	2.462	Down
hsa‐miR‐1303	chr5	4.387	Down
hsa‐miR‐191‐3p	chr3	2.815	Down
hsa‐miR‐223‐3p	chrX	2.857	Down
hsa‐miR‐1587	chrX	2.943	Down
hsa‐miR‐4286	chr8	7.635	Down
hsa‐miR‐6829‐5p	chr3	3.933	Down
hsa‐miR‐1290	chr1	6.797	Down
hsa‐miR‐2276‐3p	chr13	3.085	Down
hsa‐miR‐6735‐5p	chr1	2.266	Down
hsa‐miR‐6794‐5p	chr19	3.041	Down
hsa‐miR‐1304‐5p	chr11	2.789	Down
hsa‐miR‐4257	chr1	2.373	Down
hsa‐miR‐5703	chr2	2.863	Down
hsa‐miR‐615‐3p	chr12	2.428	Down
hsa‐miR‐377‐3p	chr14	2.498	Down
hsa‐miR‐4738‐3p	chr17	2.559	Down
hsa‐miR‐6076	chr14	2.436	Down
hsa‐miR‐6073	chr11	2.307	Down
hsa‐miR‐6739‐5p	chr1	2.279	Down
hsa‐let‐7d‐3p	chr9	3.305	Down
hsa‐miR‐1277‐3p	chrX	2.360	Down
hsa‐miR‐4734	chr17	2.329	Down
hsa‐miR‐1224‐5p	chr3	2.611	Up
hsa‐miR‐7641	chr14	2.216	Up
hsa‐miR‐190a‐5p	chr15	2.596	Up
hsa‐miR‐3667‐5p	chr22	2.224	Up
hsa‐miR‐7975	chr19	4.649	Up
hsa‐miR‐6885‐5p	chr19	2.868	Up
hsa‐miR‐1255a	chr4	2.605	Up
hsa‐miR‐8063	chr15	2.471	Up
hsa‐miR‐6808‐5p	chr1	2.006	Up

To validate the data obtained from the miRNA microarray, we examined three of the differentially expressed miRNAs in NSCLC A549 and H460 cells using qRT‐PCR. Relative expression levels of miR‐424‐3p and miR‐377‐3p were down‐regulated (Figure 3B, C), and miR‐1224‐5p was up‐regulated (Figure 3D) in A549 and H460 cells with baicalein treatment at different concentrations, which is consistent with the results from the miRNA microarrays. Thus, we confirmed that the microarray data were reliable and that miR‐424‐3p was down‐regulated by baicalein treatment.

### 3.4 PTEN was identified as a target gene of miR‐424‐3p

**Figure 4:**
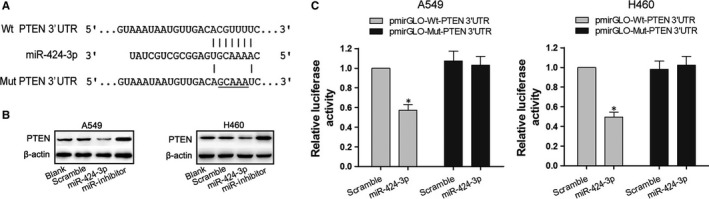
PTEN was identified as a target gene of miR‐424‐3p. (A) PTEN mRNA 3′UTR was shown to have a complementary pairing sequence with miR‐424‐3p. (B) miR‐424‐3p mimics, inhibitor and scramble were transfected into A549 and H460 cells for 48 h, and Western blotting was performed to detect the effects of miR‐424‐3p on PTEN protein expression. (C) Dual‐luciferase reporter assay was used to determine the relative luciferase activity of A549 and H460 cells cotransfected with miR‐424‐3p mimics and pmirGLO‐Wt‐PTEN 3′UTR. *P < .05

We suggested that miR‐424‐3p may mediate the effects of baicalein on the PTEN pathway according to the above results. Thus, we predicted the putative targets to explore probable biological functions of miR‐424‐3p using prediction software. A complementary pairing area exists in sequences of PTEN mRNA and miR‐424‐3p indicating PTEN mRNA 3′UTR as a target of miR‐424‐3p (Figure 4A). Therefore, we transfected miR‐424‐3p mimics, inhibitor and scramble into A549 and H460 cells for 48 hours. Western blotting revealed that the expression of PTEN was down‐regulated in cells transfected with miR‐424‐3p mimics, where it was up‐regulated in cells transfected with miR‐424‐3p inhibitor, compared to Blank cells (Figure 4B). This data indicate that PTEN may be regulated by miR‐424‐3p. We then made a point mutation of the miRNA binding site and carried out the double luciferase reporter assay to verify whether PTEN is a target gene of miR‐424‐3p. As shown in Figure 4C, mutation of the specific binding site abolished the miR‐424‐3p mimics effects on decreasing relative luciferase activity when comparing to wild‐type PTEN. This result was consistent in both A549 and H460 cells. Therefore, the luciferase reporter assay revealed that miR‐424‐3p down‐regulated the expression of PTEN by targeting the 3′UTR of PTEN mRNA.

### 3.5 Down‐regulation of miR‐424‐3p or treatment with baicalein similarly affects cell proliferation, apoptosis and cisplatin sensitivity in A549 and H460 cells

**Figure 5:**
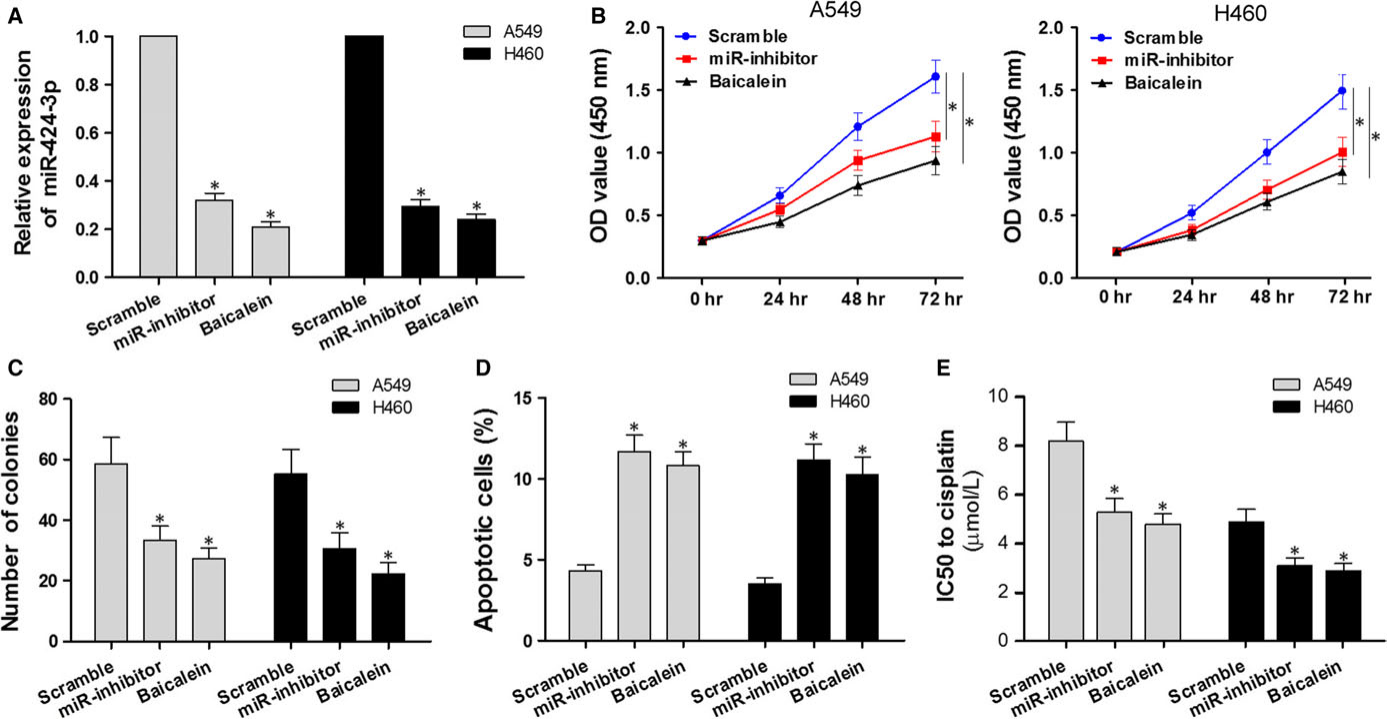
Effects of miR‐424‐3p down‐regulation or baicalein treatment on cell proliferation, apoptosis and cisplatin sensitivity in A549 and H460 cells. (A) A549 and H460 cells were treated with 40 μmol/L baicalein or transfected with miR424‐3p mimics/scramble. Expression levels of miR‐424‐3p were examined via qRT‐PCR. (B) CCK‐8 was performed to examine the effects on cell proliferation in the three groups at 0‐72 h. (C) Clone formation assay was used to detect colony number in the three groups at 24 h. (D) Annexin V‐FITC/PI double staining and flow cytometry were used to detect apoptotic cells in the three groups at 24 h. E, IC50 of cisplatin was calculated according to the OD values of cells in the three groups at 24 h when cotreated with different concentrations (0, 2, 4, 8, 16, 32 μmol/L) of cisplatin, using the CCK‐8 assay. *P < .05

To evaluate the effects of miR‐424‐3p knockdown or baicalein treatment on A549 and H460 cells, we tested three groups: cells transfected with miR‐424‐3p inhibitor, cells transfected with miR‐424‐3p scramble or cells treated with 40 μmol/L baicalein. As shown in Figure 5A, cells transfected with miR‐424‐3p inhibitor or treated with baicalein showed significant down‐regulation of the relative expression of miR‐424‐3p when compared to scramble cells. In addition, down‐regulation of miR‐424‐3p or treatment with baicalein consistently decreased cell viability (Figure 5B), decreased colony number (Figure 5C), increased cell apoptosis (Figure 5D) and reduced the IC50 of cisplatin (Figure 5E). Therefore, this study confirmed the biological effects of miR‐424‐3p down‐regulation and treatment with baicalein in A549 and H460 cells, indicating that baicalein may exert its effects on NSCLC cells via regulation of miR‐424‐3p.

### 3.6 Overexpression of miR‐424‐3p or silencing of PTEN attenuates the effects of baicalein on A549 and H460 cells

**Figure 6:**
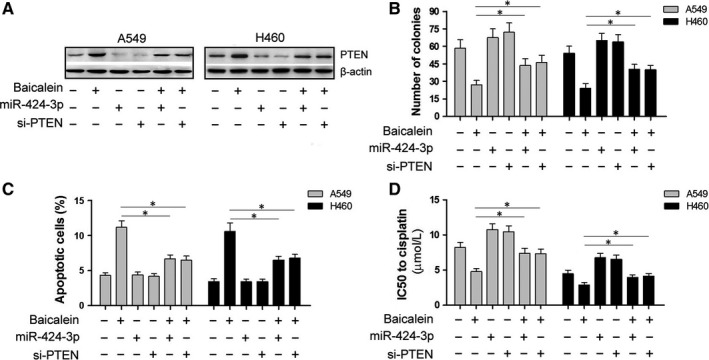
Overexpression of miR‐424‐3p or silencing of PTEN attenuates the effects of baicalein on A549 and H460 cells. Six groups were designed to determine the pathway of baicalein activity in A549 and H460 cells: Blank, baicalein treatment alone, miR‐424‐3p transfection alone, si‐PTEN transfection alone, baicalein with miR‐424‐3p transfection and baicalein with si‐PTEN transfection. (A) Expression levels of PTEN protein were detected in the six groups via Western blot. (B) Colony number was determined in the six groups via clone formation assay. (C) Apoptosis in cells was detected via Annexin V‐FITC/PI double staining and flow cytometry. (D) IC50 of cisplatin was calculated according to the OD value of cells when cotreated with different concentrations of cisplatin via CCK‐8 assay. *P < .05

We sought to further verify the regulatory pathway of baicalein/miR‐424‐3p/PTEN. Cells were treated with baicalein and transfected with either miR‐424‐3p mimics or si‐PTEN to either overexpress miR‐424‐3p or knock down PTEN expression. As shown in Figure 6A, when compared to Blank cells, PTEN protein expression was down‐regulated in miR‐424‐3p mimics and si‐PTEN‐transfected cells, and up‐regulated in baicalein‐treated cells. However, the up‐regulation of PTEN by baicalein was partially attenuated by miR‐424‐3p mimics or si‐PTEN transfection in A549 and H460 cells. Similar effects of miR‐424‐3p overexpression or PTEN silencing were observed in the proliferation, apoptosis and cisplatin sensitivity assays in A549 and H460 cells; these effects of baicalein were partially attenuated by transfection with miR‐424‐3p mimics or si‐PTEN (Figure 6B,C,D). These results suggest that baicalein inhibits proliferation, promotes apoptosis and increases cisplatin sensitivity in A549 and H460 cells via down‐regulation of miR‐424‐3p and up‐regulation of PTEN.

## 4 DISCUSSION

Baicalein is one of major flavonoids found in *Scutellaria baicalensis*. Previous studies have indicated that baicalein inhibits cell growth and metastasis and increases the sensitization of chemotherapeutic drugs via various pathways.^9^, ^10^, ^23^, ^42^, ^43^, ^44^ However, few studies have reported changes in miRNA expression. In this study, we performed a microarray analysis to determine the differential expression of miRNAs following treatment with baicalein and the potential mechanisms underlying the observed effects.

First, we showed that baicalein exhibits cytotoxic activity in NSCLC A549 and H460 cells, where only slight toxicity was observed in NHBE cells. Similar results were observed in hepatocellular carcinoma cells and normal liver cells,^14^ suggesting that baicalein is not cytotoxic to non‐cancer cells; therefore, the clinical application of baicalein may be feasible. Baicalein also inhibited cell growth, induced apoptosis and increased sensitivity to cisplatin in A549 and H460 cells. We also demonstrated that baicalein may mediate the PTEN/PI3K/Akt pathway, which is consistent with results of previous studies.^22^, ^33^, ^45^ Further, microarray analysis of miRNAs in cells treated with baicalein revealed a significant down‐regulation in the expression of miR‐424‐3p, which may interact with PTEN mRNA. miR‐424 was reported to regulate tumour growth and apoptosis in some studies. Zhang et al^46^ found that miR‐424 could decrease the sensitivity of cancer cells such as HCT116 and A375 to doxorubicin and etoposide, and inhibition of miR‐424 could enhance apoptosis and increase the sensitivity of cancer cells to doxorubicin; while Oneyama et al^47^ confirmed that down‐regulation of miR‐424/503 was associated with Rictor up‐regulation in colon cancer tissues, resulting in promotion of tumour growth and invasion. Besides, miR‐424 was reported that loss of miR‐424‐3p conferred chemoresistance through targeting YAP1 in non‐small‐cell lung cancer.^48^ Our study showed the effects of miR‐424‐3p on NSCLC cells. The dual‐luciferase reporter assay demonstrated that PTEN is a target gene of miR‐424‐3p, and down‐regulation of miR‐424‐3p or treatment with baicalein resulted in similar effects on cell proliferation, apoptosis and cisplatin sensitivity in A549 and H460 cells. This result suggests miR‐424‐3p exerts its effects through the PTEN pathway. Overexpression of miR‐424‐3p or silencing of PTEN partially attenuated the effects of baicalein on A549 and H460 cells, suggesting that baicalein induced down‐regulation of miR‐424‐3p and up‐regulation of PTEN. In summary, our findings demonstrate that baicalein may inhibit A549 and H460 cell growth and increase cisplatin sensitivity via down‐regulation of miR‐424‐3p and targeting of the PTEN/PI3K/Akt pathway. However, it is highly likely that other pathways may be involved, and future studies will investigate alternative pathways of action.

## 5 CONCLUSION

Baicalein inhibits cell growth and increases cisplatin sensitivity of A549 and H460 cells via down‐regulation of miR‐424‐3p and targeting of the PTEN/PI3K/Akt pathway. The cytotoxic effects of baicalein are dose‐independent in NSCLC A549 and H460 cells, where only a slight toxicity was observed in normal human bronchial epithelial cells. Baicalein has potential as an adjuvant therapy in NSCLC.

## CONFLICT OF INTEREST

The authors confirm that there is no conflict of interest.

## AUTHORS' CONTRIBUTIONS

GJZ and CYL designed the study; CYL, HQW, SSC and HL carried out part of experiments; and CYL wrote the manuscript and performed the statistical analysis. All authors have approved the final manuscript.
